# Mechanisms of Puerariae Lobatae Radix in regulating sebaceous gland secretion: insights from network pharmacology and experimental validation

**DOI:** 10.3389/fphar.2024.1414856

**Published:** 2024-07-24

**Authors:** Du Yijie, Zhao Siqi, Huang Ruiyin, Shi YuJing, Meng Hong, Dong Yinmao, Yang Tao, Luo Changyong

**Affiliations:** ^1^ Beijing Technology and Business University, Beijing Technology & Business University, Beijing, China; ^2^ R&D Center, Beijing Academy of TCM Beauty Supplements, Beijing, China; ^3^ Dermatology, Dongfang Hospital, Beijing University of Chinese Medicine, Beijing, China; ^4^ Institute of Chinese Materia Medica, China Academy of Chinese Medical Sciences, Beijing, China; ^5^ Traditional Chinese Medicine Department, Beijing Shijitan Hospital, Capital Medical University, Beijing, China; ^6^ Infectious Fever Center, Dongfang Hospital, Beijing University of Chinese Medicine, Beijing, China

**Keywords:** Puerariae Lobatae Radix, sebaceous gland regulation, network pharmacology, lipid metabolism, animal experiments

## Abstract

**Objective:**

This research aims to explore how Puerariae Lobatae Radix regulates sebaceous gland secretion using network pharmacology, and validate its effects on important targets through animal studies.

**Methods:**

This study utilized UPLC-EQ-MS to analyze Puerariae Lobatae Radix extract and identify potential bioactive compounds. Predicted targets of these compounds were obtained from the Swiss Target Prediction database, while targets associated with sebaceous gland secretion were obtained from the GeneCards database. Common targets between the databases were identified and a protein-protein interaction (PPI) network was established using the STRING platform. The PPI network was further analyzed using Cytoscape software. Pathway enrichment analysis was performed using Reactome, and molecular docking experiments targeted pivotal pathway proteins. Animal experiments were then conducted to validate the regulatory effects of the primary active compounds of Puerariae Lobatae Radix on key pathway proteins.

**Results:**

This research identified 17 active compounds in Puerariae Lobatae Radix and 163 potential targets associated with the regulation of sebum secretion. Pathway enrichment analysis indicates that these targets may modulate lipid metabolism pathways through involvement in peroxisome proliferator-activated receptor α, SREB, steroid metabolism, and arachidonic acid metabolism pathways. Molecular docking analysis demonstrates that puerarin and daidzein show favorable binding interactions with key targets in these pathways. Animal experiments demonstrated that the administration of Puerariae Lobatae Radix resulted in a significant reduction in the area of sebaceous gland patches compared to the control group. Histological analysis revealed notable alterations in the structure of sebaceous glands, including reductions in size, thickness, and density. Furthermore, the expression levels of TG, DHT, and IL-6 were significantly decreased in the Puerariae Lobatae Radix group (*p* < 0.05), and immunoblotting indicated a significant decrease in the expression of PPARG and ACC1 (*p* < 0.05).

**Conclusion:**

This study demonstrates that Puerariae Lobatae Radix can regulate skin lipid metabolism by targeting multiple pathways. The primary mechanism involves inhibiting sebaceous gland growth and reducing TG secretion by modulating the expression of PPARG and ACC1. Puerarin and Daidzein are identified as key bioactive compounds responsible for this regulatory effect. These findings highlight the therapeutic potential of Puerariae Lobatae Radix in addressing sebaceous gland-related conditions.

## 1 Introduction

Sebaceous glands are specialized skin appendages associated with hair follicles, they play a major role in skin homoeostasis (Zouboulis et al., 2022). The main function of sebaceous glands is to secrete lipids, the human skin lipid content generally accounts for 3.5%–6.0% of the total skin weight, with the lowest being only 0.3% and the highest reaching up to 10%. Clinically, skin is roughly categorized into several types based on the surface lipid content: oily skin, dry skin, neutral skin, and combination skin, among others. Normal sebum secretion is of significant importance for maintaining the normal skin barrier function and regulating body temperature (Lupi, 2008; Hunt et al., 2017). Irregular sebum secretion has the potential to induce the proliferation of keratinocytes, exacerbate excessive proliferation and incomplete keratinization of keratinocytes, prompt the overexpression of inflammatory cytokines such as IL6, and ultimately result skin inflammation and lipid synthesis in sebaceous gland cells. (Zouboulis et al., 2021). Furthermore, the secretion of sebum is intricately linked to sex hormones, with dihydrotestosterone (DHT) serving as a potent androgen. DHT plays a crucial role not only in sebum production, but also in the release of pro-inflammatory cytokines by sebaceous gland cells. Research indicates that DHT facilitates the excessive proliferation of sebaceous gland cells and sebum production by synergizing with peroxisome proliferators-activated receptors (PPARs), ultimately resulting in skin damage (Rosenfield et al., 1998; Lopes et al., 2021). The sebaceous glands and their main products, sebaceous gland lipids, have made significant contributions in skin physiology and pathology and can be regarded as important targets for the treatment of various skin diseases in the future.

Puerariae Lobatae Radix, a traditional medicinal plant, has a rich history of use in East Asia and Southeast Asia. It contains various chemical components, primarily including isoflavones, triterpenoids, and glycosides. Among them, the most abundant and extensively studied components in Puerariae Lobatae Radix are isoflavones, including Puerarin and Daidzein. Puerariae Lobatae Radix, from the legume family, is the dried root of wild Puerariae Lobatae Radix, the roots of Pueraria lobata have been reported to prevent cardiovascular disease, control diabetes and have strong antioxidant, anti-inflammatory as well as anti-hypertensive activities (Wong et al., 2011). In recent years, multiple studies have shown that isoflavones in Puerariae Lobatae Radix exhibit various pharmacological effects, such as improving lipid metabolism (Wong et al., 2011) and reducing lipid peroxidation to enhance cardiovascular and cerebrovascular protection (Wong et al., 2011).

**FIGURE 1:**
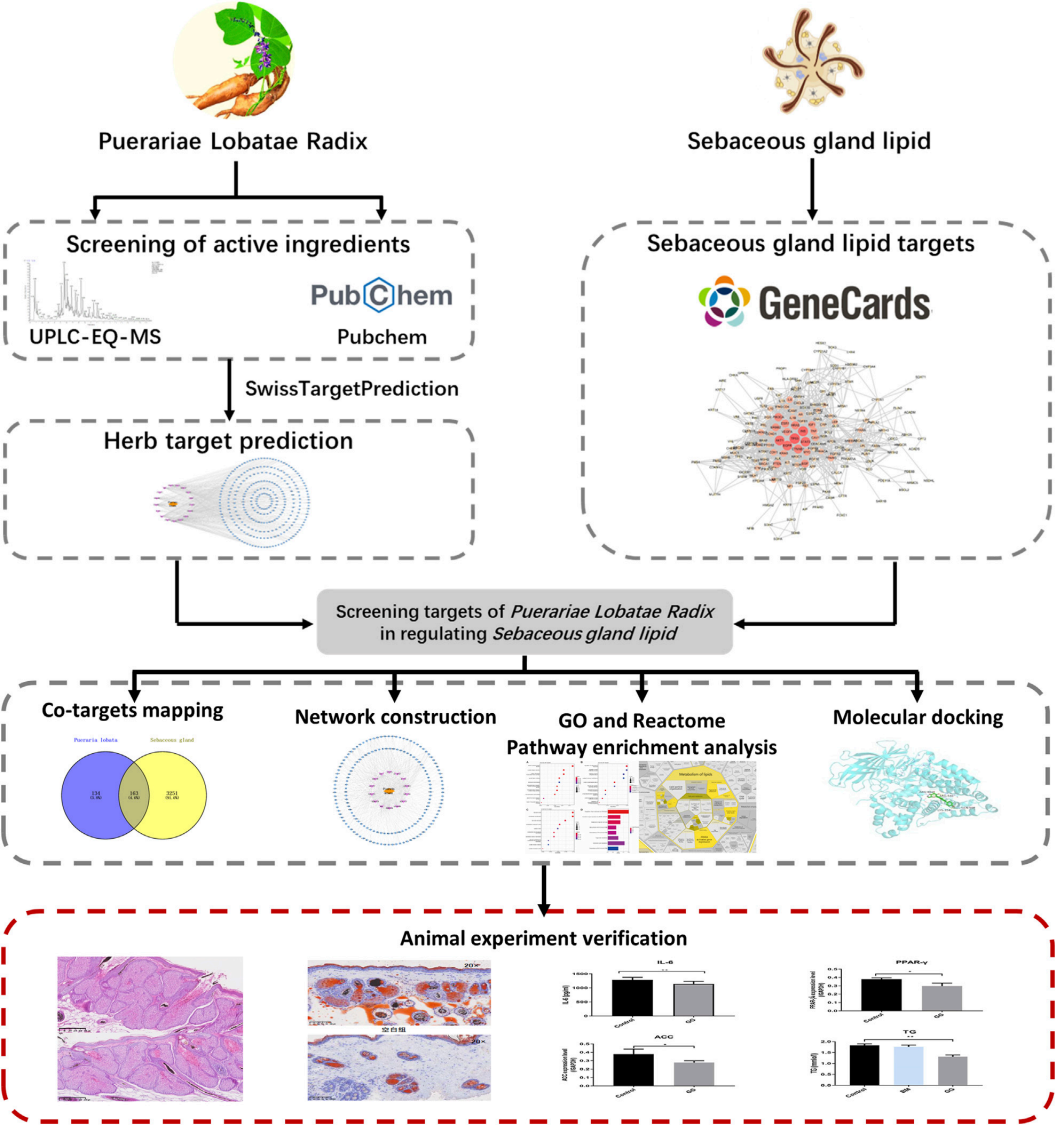
Research workflow diagram.

In recent years, Puerariae Lobatae Radix has attracted certain attention and research in the field of skin diseases, it can be used as a skin whitening agent with antioxidant and anti-melanogenesis activity (Gao et al., 2022). While current research highlights the pivotal roles of compounds such as Puerarin and Daidzein in Puerariae Lobatae Radix, there remains a dearth of studies investigating its potential in improving lipid metabolism through transdermal application. This study employed network pharmacology research methods to identify the key pathways and targets involved in the regulation of sebaceous gland lipids by Puerariae Lobatae Radix. Molecular docking methods were utilized to simulate the interaction between the main components of Puerariae Lobatae Radix and its key targets. The effect of Puerariae Lobatae Radix on the pathological changes of sebaceous gland tissue in golden hamsters was evaluated using oil red O staining in the animal experiment section. Furthermore, ELISA and Western blotting were employed to assess the regulatory impact of Puerariae Lobatae Radix on pivotal proteins involved in sebum metabolism. The objective of this investigation is to elucidate the principal pathways and critical targets of Puerariae Lobatae Radix in modulating sebaceous gland secretion, offering insights for subsequent comprehensive studies and clinical utilization. The schematic representation of the experimental process is illustrated in Figure 1.

## 2 Methods

### 2.1 Screening of active components in Puerariae Lobatae Radix

The Puerariae Lobatae Radix extract underwent a comprehensive analysis using the UPLC-EQ-MS method. UPLC-EQ-MS was employed to acquire chromatographic peak primary and secondary mass spectrometry data. Thermo Scientific Xcalibur software was utilized to compute high-resolution accurate relative molecular masses, enabling swift speculation of the corresponding molecular formulae of compounds. This facilitated comparisons with secondary fragments documented in literature, aiding in the inference and confirmation of compound composition. This process provided a detailed description of the substance composition and its contents within the sample. Specific operational procedures are as follows: Sample Pre-treatment: Accurately weigh the homogenized sample and place it in a 2 mL centrifuge tube. Add 1 mL of 70% methanol and a 3 mm steel ball. Utilize an automatic sample grinder to shake and crush the sample for 3 min. After cooling, it undergoes ultrasonication at a low temperature for 10 min. Centrifuge at 12,000 rpm for 10 min at 4°C. Collect the supernatant and dilute, then add 10 μL of a 100 μg/mL internal standard. The resulting solution is filtered through a 0.22 μm PTFE filter head before injection into the chromatograph for detection. Chromatographic conditions were as follows: Zorbax Eclipse C18 column (100 mm × 2.1 mm, 1.8 μm) was utilized. The mobile phase consisted of 0.1% formic acid in water (A) and 100% acetonitrile (B). Gradient elution was performed as follows: 0–2 min, 95% (A); 2–7 min, 70% (A); 7–14 min, 22% (A); 14–20 min, 5% (A); 20–25 min, 95% (A). The volume flow rate was set at 0.3 mL/min, with a sample size of 2.0 μL. The column temperature was maintained at 30°C, while the automatic injector temperature was set to 4°C. For MS conditions, an electrospray ion source (ESI) was employed with ionization in both positive and negative modes. The spray voltage was set at 3.5 kV, while the capillary temperature was maintained at 330°C. Primary mass spectrometry full scanning was conducted at a resolution of 120,000, covering a scanning range of m/z 100–1,500 over a scanning time of 40 min. This methodology ensures an accurate and comprehensive analysis of the constituents present in the Puerariae Lobatae Radix extract.

### 2.2 Prediction of Puerariae Lobatae Radix and sebaceous gland lipid targets

The active constituents of Puerariae Lobatae Radix were imported into the PubChem database (https://pubchem.ncbi.nlm.nih.gov/) to obtain the SMILES structures of each active ingredient. Using these SMILES structures, target predictions were conducted on the Swiss Target Prediction platform (http://www.swisstargetprediction.ch/), selecting targets with a Probability score greater than 0. The Gene Cards database (https://www.genecards.org/) was searched using the keyword “Sebaceous gland lipids” to identify targets related to sebaceous gland lipid metabolism.

### 2.3 Mapping of drug-component-shared targets and construction of protein interaction network

Through the above processes, we identified the active constituents and their corresponding targets from Puerariae Lobatae Radix, as well as the targets related to sebaceous gland lipid metabolism. Mapping the two sets of data allowed us to identify shared targets, which are potential points where Puerariae Lobatae Radix might regulate sebaceous gland lipid metabolism. A “Drug-Component-Shared Target” network was constructed and visualized using Cytoscape 3.7.1 software. The shared targets were then imported into the STRING database (https://string-db.org/) to derive the protein-protein interaction (PPI) network, which was also visualized using Cytoscape. Furthermore, the Maximum clique Centrality algorithm (MCC) of the CytoHubba plugin in Cytoscape was utilized to conduct topological analysis on the PPI network, extracting key targets as critical points where Puerariae Lobatae Radix might regulate sebaceous gland lipid metabolism.

### 2.4 Biological enrichment analysis

This study conducted biological enrichment analyses on shared targets between the drug and disease, which included both GO enrichment analysis and Reactome pathway analysis. GO enrichment can be broken down into three facets: Molecular Function (MF), Biological Process (BP), and Cellular Component (CC). The GO enrichment was completed using the R package “clusterprofiler”. The Reactome pathway analysis was conducted on the Reactome online platform (https://reactome.org/). Enrichment results with a threshold value of *p* < 0.05 were selected, and the top-ranked entries were visualized using *R* software.

### 2.5 Molecular docking

According to the Reactome pathway enrichment results, the pathways most related to sebaceous gland lipid metabolism were selected, and the corresponding active components were subjected to molecular docking. The sdf format of the traditional Chinese medicine active components was exported from the PubChem database and converted to mol2 format. Afterward, Autodock tools software was used to add full hydrogen to the small molecules, set them as ligands, detect torsional keys, select torsional keys, and so forth, saving them in pdbqt format. The pdb format file of the large molecular protein was exported from the RCSB PDB database (http://www.rcsb.org). Pymol software was used to remove water and ligands from the protein. Autodock tools software was further used to add full hydrogen to the protein, exporting it in pdbqt format. Autodock vina software was run to perform molecular docking, and the docking effect between the active components and the core targets was evaluated by the binding energy of the ligand and the receptor. Docking states with binding energies of < −7.0 Kcal·mol-1 were considered good (Zhu et al., 2024), and the molecular docking results were visualized using Pymol software.

### 2.6 Animal experiment verification

#### 2.6.1 Experimental materials

Test Drugs: Freeze-dried Puerariae Lobatae Radix powder, batch number: wc20230,105, characteristics: yellow gel. The sample was provided by Beijing Academy of TCM Beauty Supplements, Beijing, Beijing. Preparation method: 100 g of Puerariae Lobatae Radix were subjected to extraction, wherein the powder was treated with a 2000 mL solution of 50% ethanol at 80°C for 2 h through hot reflux. Subsequently, the solution was concentrated and freeze-dried, resulting in the production of 19.38 g of pueraria lyophilized powder. The yield of pueraria lyophilized powder was calculated to be 19.38%. Received date: 6 January 2023. Storage conditions: Stored in a cool, dry, and dark place.

Reagents: The experimental procedures utilized a range of materials and reagents, sourced from different companies. Triglyceride (TG) Content Detection Kit (Solabio, catalog number BC0625) and Dihydrotestosterone (DHT) ELISA Kit (Solabio, catalog number SEKSM-0030) were used for triglyceride and dihydrotestosterone analysis, respectively. The Rat Interleukin-6 (IL-6) ELISA Kit (Jiangsu Enzyme Immunoassay Co., Ltd., catalog number MM-0190R2) was employed for IL-6 measurement. Antibodies such as ACC1 (Proteintech, catalog number 67373-1-Ig) and PPAR-γ (Proteintech, catalog number 16643-1-AP) were used for specific protein detection. Additionally, the GAPDH Mouse Monoclonal Antibody (Immunoway, catalog number YM3029) was utilized. Staining reagents included Hematoxylin (BDHM, catalog number BDHM-H1) and Oil Red O (Solabio, catalog number G1262). The contributions of these companies in providing the necessary materials and reagents are greatly acknowledged.

Experimental Animals: 30 SPF-grade male golden hamsters, weighing 110–130 g, purchased from Beijing Weitonglihua Experimental Animal Technology Co., Ltd. The experimental site was provided by Beijing Yongxinkangtai Technology Development Co., Ltd.’s animal experiment center, with an indoor temperature of 21–23°C, humidity of 52%–60%, ventilation, and separate cage breeding. They had unlimited access to food and water and were acclimatized for 1 week before the experiment. The animal experiments were approved by the Experimental Animal Welfare and Ethics Committee (batch number: YXKT2022L027).

#### 2.6.2 Experimental methods

##### 2.6.2.1 Animal grouping and administration

Based on weight, test animals were randomly divided into three groups: blank group, matrix group, and Puerariae Lobatae Radix freeze-dried powder group, with 10 in each group. Distilled water, gel matrix, and Puerariae Lobatae Radix gel were respectively applied to the sebaceous gland patches of the animals in each group once daily for four consecutive weeks. Puerariae Lobatae Radix gel is prepared by adding 0.6 g of freeze-dried pueraria powder into the gel matrix formula to produce 100 g of Puerariae Lobatae Radix gel. The gel is then applied externally at a dose of 0.2 g/tablet per day.

##### 2.6.2.2 Sebaceous gland patch measurement

Before the first administration and weekly thereafter, the hair on both sides of the back hair of 10 hamsters in each group was shaved to fully expose the sebaceous gland patch. Using calipers, the maximum transverse diameter (DT) and the maximum longitudinal diameter (DL) of the sebaceous gland patch were measured. The sebaceous gland area (mm^2) = π × (DT/2) × (DL/2).

##### 2.6.2.3 Histopathology

24 h after the final administration, tissue from the left sebaceous gland patch of 5 hamsters from each group was taken. After fixation in 4% paraformaldehyde, the tissues were embedded in paraffin, sectioned, and stained with hematoxylin and eosin (HE) for microscopic observation.

##### 2.6.2.4 Oil red O staining of sebaceous gland tissue

24 h after the last administration, tissue from the right sebaceous gland patch of 5 hamsters from each group was taken. After freezing section fixation, the tissue was stained with Oil Red O and hematoxylin. Lipid droplets appeared orange-red under the microscope.

##### 2.6.2.5 Triglyceride (TG) detection

Tissue samples from the left sebaceous gland patches of 5 hamsters per group were collected 24 h post-administration. The tissue was homogenized in PBS to create a 20 mg/mL tissue suspension. After centrifuging at 3,000 rpm for 20 min, the supernatant was collected for TG and inflammatory factor measurements. TG was detected using biochemical methods (specific steps are detailed in the instructions). Results were analyzed using one-way ANOVA, with *p* < 0.05 considered statistically significant.

##### 2.6.2.6 Inflammatory factor ELISA detection of DHT and IL-6

24 h after the final administration, five specimens of sebaceous gland tissue from the left side of each group of golden hamsters were collected. The tissue was homogenized in PBS to obtain a 20 mg/mL tissue suspension. The suspension was centrifuged at 3,000 rpm for 20 min, and the supernatant was collected for measurement of triglycerides (TG) and inflammatory factors. The ELISA assay for inflammatory factors followed the provided instructions. The assay steps were as follows: Standard Sample Preparation: Various concentrations of standard samples (50 μL each) were added to designated wells. Sample Addition: Blank wells were prepared as controls, while the sample wells received 40 μL of sample dilution buffer followed by 10 μL of the test sample (resulting in a 5-fold dilution). Enzyme Addition: Enzyme reagent (100 μL) was added to each well, except for the blank wells. Incubation: The plate was sealed with a cover film and incubated at 37°C for 60 min. Washing: The cover film was removed, and the wells were washed with washing solution; this process was repeated five times. Color Development: A mixture of color reagent A (50 μL) and color reagent B (50 μL) was added to each well, and the plate was gently shaken before being incubated at 37°C in darkness for 15 min. Termination: Stop solution (50 μL) was added to each well, resulting in a color change from blue to yellow. Measurement: The absorbance (OD values) of each well was measured at a wavelength of 450 nm using the blank well as reference point. The results underwent analysis using one-way analysis of variance; significance level *p* < 0.05 indicated statistical significance.

##### 2.6.2.7 Western blotting detection of PPARG and ACC1

Protein Extraction: The tissues were homogenized using a Fluka electric tissue homogenizer at 15,000 rpm for 10 s, with 10-s intervals, for a total of three cycles. The homogenization was performed in pre-cooled RIPA protein extraction reagent containing proteinase inhibitor (phosphatase inhibitor was added for phosphorylated proteins). Prior to extraction, 1 mM PMSF stock solution was added to achieve the final concentration. The tissue-to-lysis buffer ratio was 1:9. After homogenization, the sample was incubated on ice for 20 min and then centrifuged at 13,000 rpm for 20 min at 4°C. The supernatant was collected and stored for further analysis. BCA Protein Quantification: BCA working solution was prepared by mixing A solution with B solution at a ratio of 50:1. BSA standards were prepared at various concentrations. The samples were diluted with PBS, and the sample-to-BCA working solution ratio was 1:8. After incubation at 37°C for 30 min or at room temperature for 60 min, the absorbance of the samples was measured at 570 nm using an ELISA reader. Protein Concentration Adjustment: The protein concentration was adjusted using RIPA buffer. The samples were treated with 5 × reducing sample buffer to achieve a final concentration of 2 mg/mL. The samples were then boiled for 5 min to denature the proteins. Western blotting for Target Proteins: Separation gels with concentrations of 8% and 12% were prepared based on the target proteins’ molecular weights, with a final gel concentration of 5%. The samples, loaded at 20 μg per lane, underwent electrophoresis using a constant voltage of 90V for the stacking gel and 160V for the separation gel. The electrophoresis was stopped based on the migration of a pre-stained protein marker. The proteins were transferred onto a nitrocellulose membrane using the wet transfer method with appropriate transfer times. After transfer, the membrane was stained with Ponceau S staining reagent to assess transfer efficiency and mark the lanes. Blocking: The membrane was immersed in 3% BSA-TBST and gently shaken for 30 min at room temperature. Primary Antibody Incubation: The primary antibodies, including SQLE (1:1,000), ACC (1:500), PPAR-γ (1:1,000), and LTB4 (1:1,000), were diluted in 3% BSA-TBST. The membrane was incubated with the primary antibodies at room temperature for 10 min, followed by overnight incubation at 4°C. On the following day, the membrane was taken out from 4°C and incubated at room temperature for 30 min. Membrane washing: The membrane was washed with TBST five times, each for 3 min. Secondary antibody incubation: The secondary antibody, goat anti-rabbit IgG (H + L) HRP, was diluted in 5% skim milk-TBST at a ratio of 1:10,000. The membrane was incubated with the secondary antibody at room temperature with gentle shaking for 40 min. Membrane washing: The membrane was washed with TBST six times, each for 3 min. After adding the ECL reagent onto the membrane, it was allowed to react for 3–5 min. Film exposure was performed for 10 s to 5 min (exposure time adjusted according to different light intensities), followed by a 2-min development and fixing. Reference protein Western blot: The membrane was washed with Stripping Buffer at 37°C for 30 min. After washing with deionized water three times, the membrane was washed with TBST three times, each for 3 min. The membrane was immersed in 3% BSA-TBST and gently shaken at room temperature for 30 min. Incubation with reference protein: The membrane was incubated with GAPDH mouse monoclonal antibody, diluted 1:20,000 in 3% BSA-TBST, at room temperature for 2 h. Membrane washing: The membrane was washed with TBST five times, each for 3 min. Secondary antibody incubation: The secondary antibody, goat anti-mouse IgG (H + L) HRP, was diluted in 5% skim milk-TBST at a ratio of 1:10,000. The membrane was incubated with the secondary antibody at room temperature with gentle shaking for 40 min. Membrane washing: The membrane was washed with TBST six times, each for 3 min. Membrane washing: The membrane was washed with TBST five times, each for 3 min. After adding the ECL reagent onto the membrane, it was allowed to react for 3–5 min. Film exposure was performed for 10 s to 5 min (exposure time adjusted according to different light intensities), followed by a 2-min development and fixing. Data processing: The exposed film was directly scanned, and the software ImageJ was used to convert the image format from JPEG to TIF. Total Lab Quant V11.5 software (Newcastle upon Tyne, United Kingdom) was used to read the integrated optical density (IOD) values of the bands. The results were expressed as the ratio of the target protein band IOD to the reference protein GAPDH band IOD. One-way analysis of variance was used to determine intergroup differences, with *p* < 0.05 indicating statistically significant differences.

## 3 Results

### 3.1 Screening results of active components in Puerariae Lobatae Radix

A total of 470 pieces of data were obtained. After deduplication of positive and negative ion modes with similar retention time and identical molecular weight, 324 pieces of data were selected. Comprehensive screening of Puerariae Lobatae Radix compounds was conducted based on literature, sample content and acquisition difficulty. By comparing Puerariae Lobatae Radix constituents in PubChem database, Puerariae Lobatae Radix constituents in Chinese medicine bank were selected. According to the relative percentage (in %) of each substance in the Puerariae Lobatae Radix sample, select the component with higher content in the Puerariae Lobatae Radix sample. Give priority to obtaining difficult and easy Puerariae Lobatae Radix ingredients.

**FIGURE 2:**
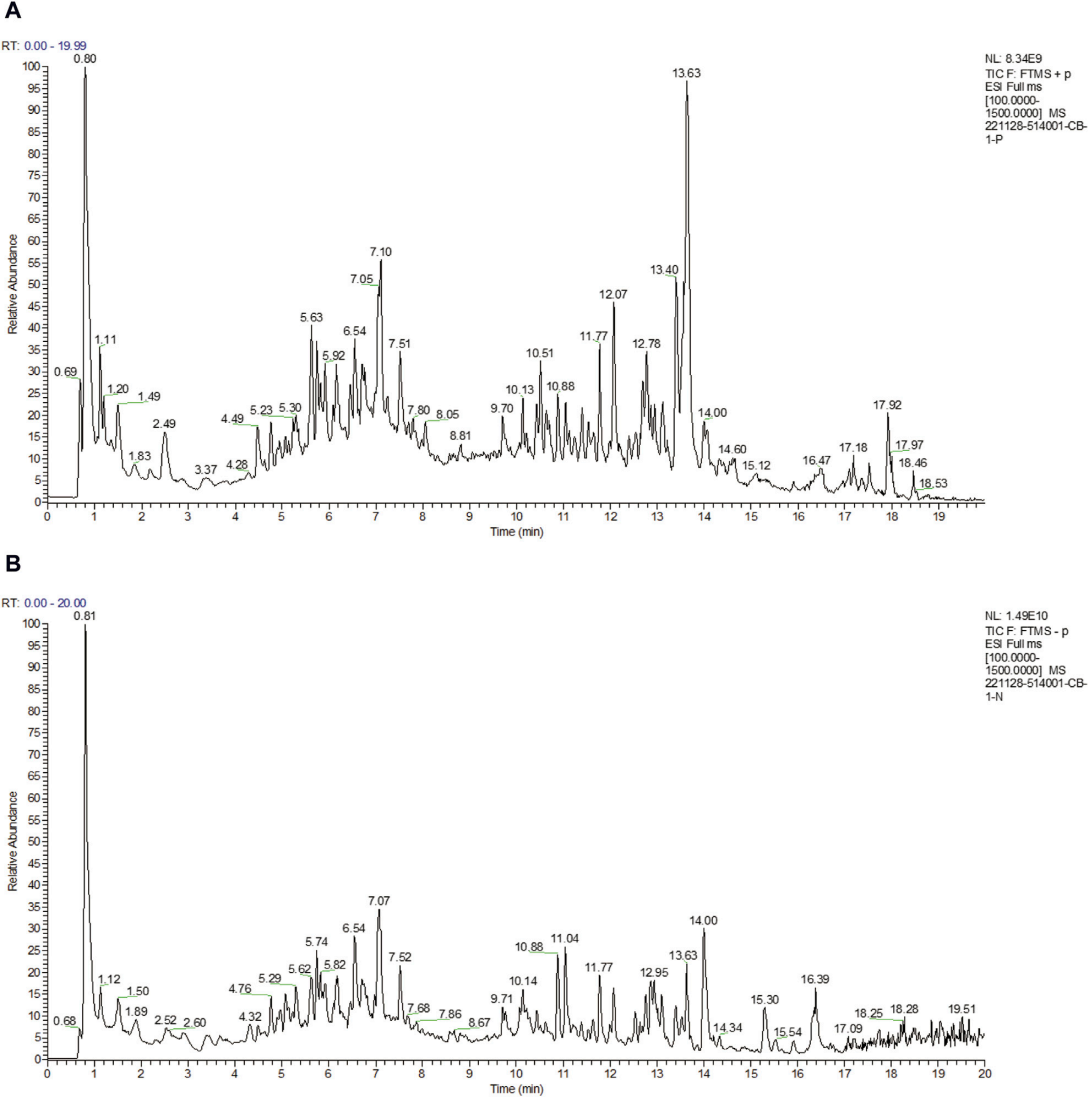
**(A)** Total ion flow diagrams in positive ion modes. **(B)** Total ion flow diagrams in negative ion modes.

A total of 17 active ingredients of Puerariae Lobatae Radix were obtained as shown in Table 1, and the total ion flow chart is shown in Figure 2.

**TABLE 1:** Information on active components of Puerariae Lobatae Radix.

Name	Formula	PubChemID	CAS	Content (%)
Puerarin	C21 H20 O9	3,681–99–0	5281807	6.716
Daidzein	C15 H10 O4	486–66–8	5281708	6.032
Ononin	C22 H22 O9	486–62–4	442,813	1.954
Formononetin	C16 H12 O4	485–72–3	5280378	1.639
Puerarin apioside	C26 H28 O13	103,654–50–8	21676217	1.420
Calycosin	C16 H12 O5	20,575–57–9	5280448	1.240
Genistein	C15 H10 O5	446–72–0	5280961	0.803
Biochanin A	C16 H12 O5	491–80–5	5280373	0.615
Coumestrol	C15 H8 O5	479–13–0	5281707	0.331
Corylin	C20 H16 O4	53,947–92–5	5316097	0.064
Daidzin	C21 H20 O9	552–66–9	107,971	0.028
Isoliquiritigenin	C15 H12 O4	961–29–5	638,278	0.028
palmitic acid	C16 H32 O2	57–10–3	985	0.016
Lupenone	C30 H48 O	1,617–70–5	92,158	0.008
Scoparone	C11 H10 O4	120–08–1	8417	0.003
Apigenin	C15 H10 O5	520–36–5	5280443	1.185
Vitexin	C21 H20 O10	3,681–93–4	5280441	3.100

### 3.2 Mapping of drug-component-shared targets and network construction

**FIGURE 3:**
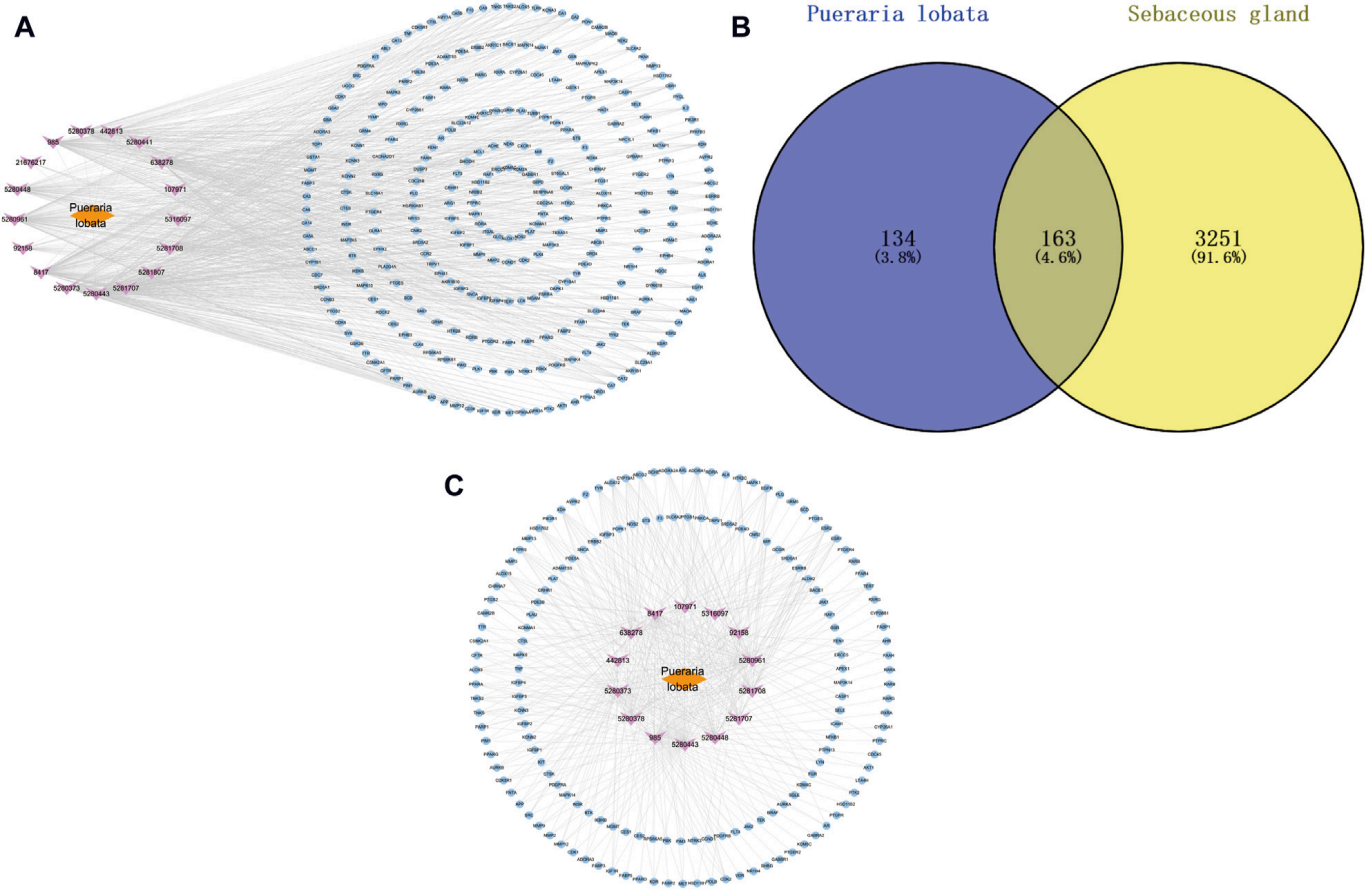
**(A)** Puerariae Lobatae Radix “active ingredients-target” network (from which 17 active ingredients of Puerariae Lobatae Radix were screened, and a total of 297 Chinese medicine targets were obtained through predictive analysis), **(B)** Venn diagram of common targets between Puerariae Lobatae Radix and sebaceous gland lipids (intersecting 3,414 sebaceous gland lipid-related targets and 297 Puerariae Lobatae Radix targets, resulting in 163 shared targets), **(C)** “Drug-ingredient-shared target” network (comprising 14 active ingredients and 163 shared targets).

Through Swiss Target Prediction, the targets of each component were predicted. After removing duplicated targets, a total of 297 traditional Chinese medicine targets were obtained. Figure 3A shows the network of various active components of Puerariae Lobatae Radix and their target actions. A total of 3,414 sebaceous gland lipid-related targets were retrieved from the GeneCards database. By mapping the 297 targets of Puerariae Lobatae Radix obtained above with sebaceous gland lipid-related targets, 163 shared targets were obtained, as shown in Figure 3B. Furthermore, the “drug-ingredient-shared target” network was constructed, as shown in Figure 3C, which contains 14 active ingredients and 163 shared targets.

### 3.3 Construction and analysis of protein-protein interaction networks

**FIGURE 4:**
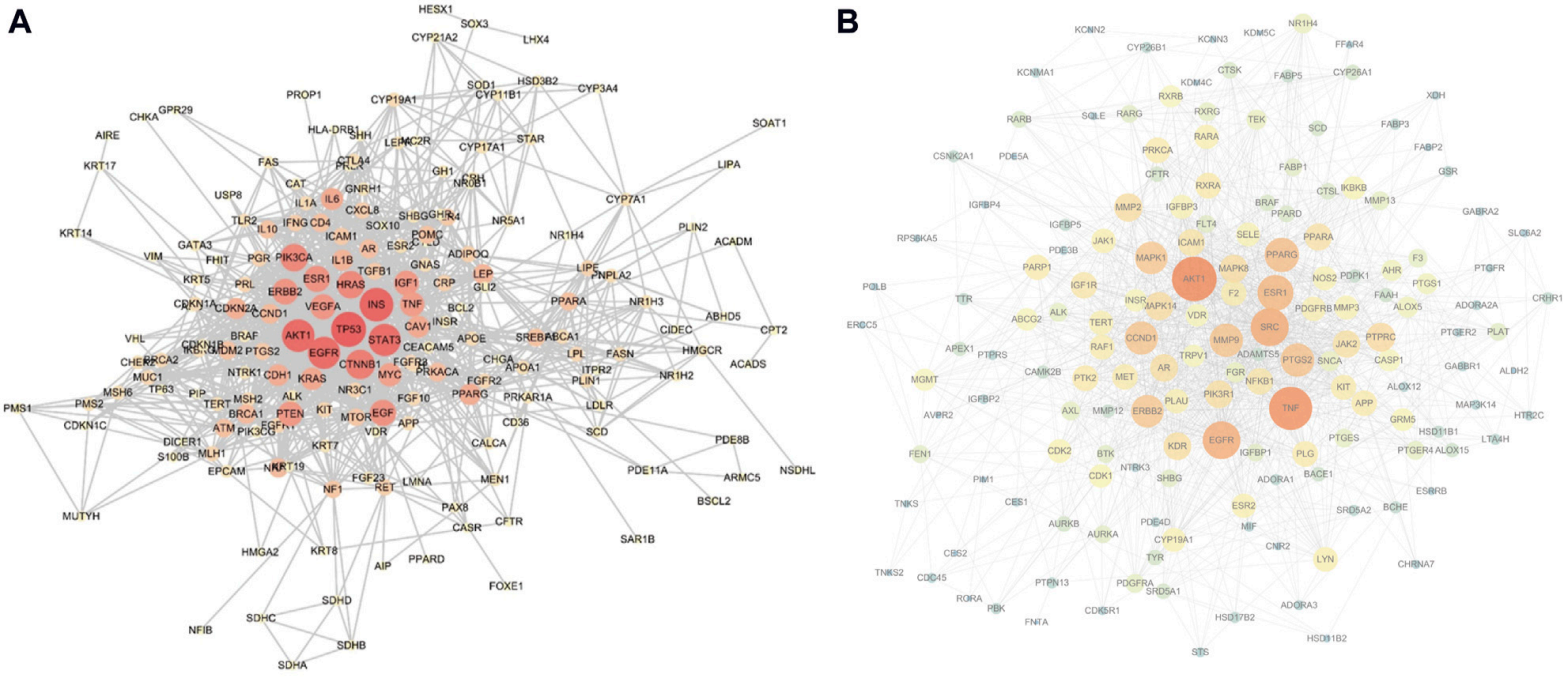
**(A)** Sebaceous gland target PPI network (containing 182 nodes and 1,184 edges; larger nodes and redder colors indicate a higher Degree value), **(B)** Shared target PPI network (containing 161 nodes and 1,368 edges; larger nodes and redder colors indicate a higher Degree value).

The top 200 targets related to the sebaceous gland were imported into the STRING platform to obtain the PPI network mapping relationship of the shared targets. The PPI network was further visualized using Cytoscape software, as shown in Figure 4A. The network contains 182 nodes and 1,184 edges, and is displayed based on the Degree value of the nodes. The larger the node and the redder the color, the larger the Degree value of the node. The top 10 targets with the highest Degree values are: TP53, INS, AKT1, STAT3, EGFR, CTNNB1, PIK3CA, HRAS, EGF, and ESR1.

The aforementioned 163 shared targets were imported into the STRING platform to obtain the PPI network mapping relationship. The PPI network was further visualized using Cytoscape software, as shown in Figure 4B. The network contains 161 nodes and 1,368 edges, and is displayed based on the Degree value of the nodes. The top 10 targets with the highest Degree values are: AKT1, TNF, SRC, EGFR, ESR1, PPARG, PTGS2, CCND1, MMP9, and ERBB2.

### 3.4 Biological enrichment analysis of shared targets

**FIGURE 5:**
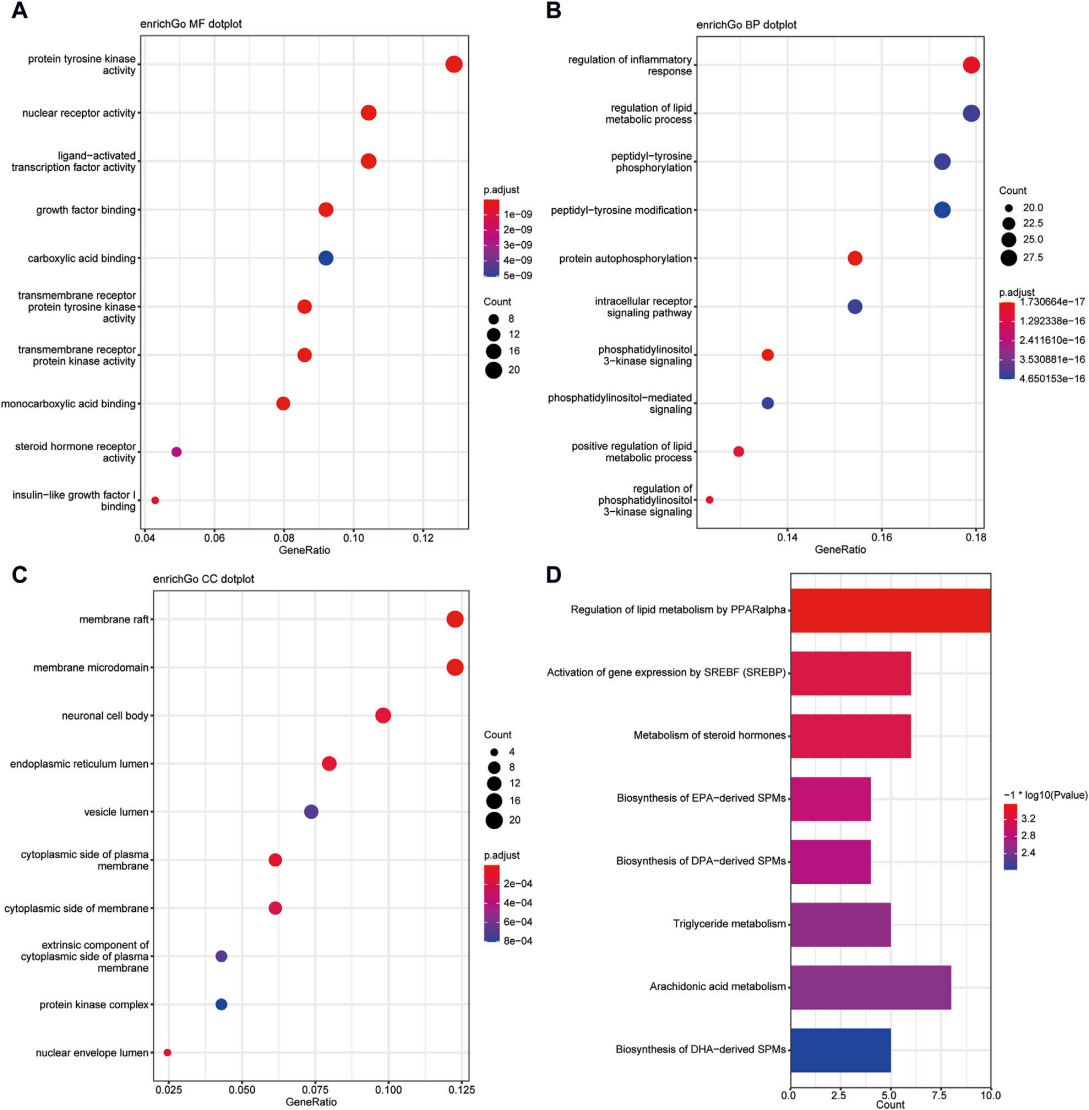
**(A)** GO- Molecular Function enrichment analysis of shared targets. **(B)** GO-Biological Process enrichment analysis of shared targets. **(C)** GO- Cellular Component enrichment analysis of shared targets. **(D)** KEGG pathway enrichment analysis of shared targets.

**FIGURE 6:**
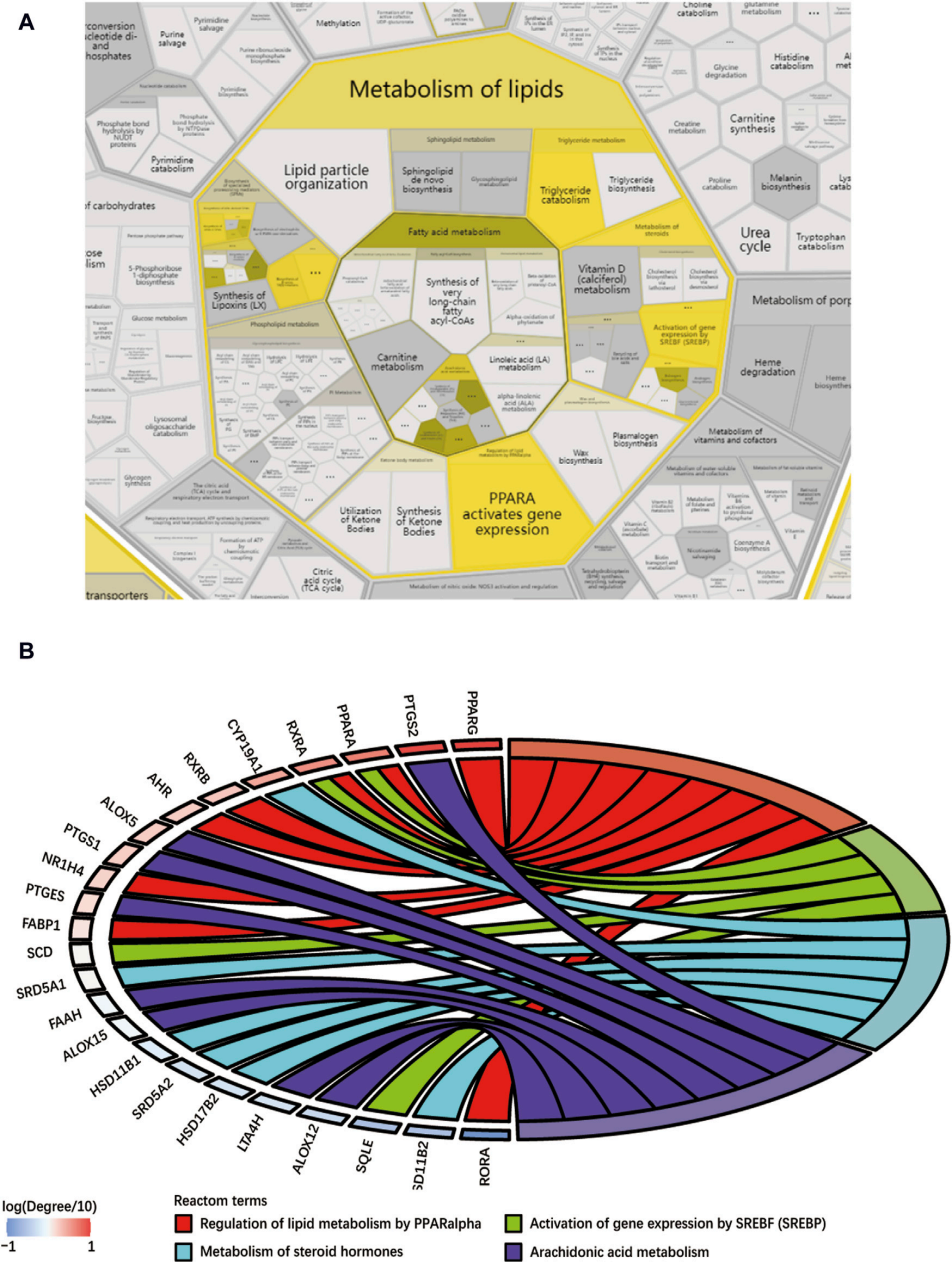
**(A)** Reactome lipid metabolism pathway enrichment diagram, **(B)** Key pathway and target chord diagram [the color blocks of the targets represent log (Degree/10)].

This study conducted a biological enrichment analysis of the 163 shared targets. GO enrichment results: MF: nuclear receptor activity, ligand-activated transcription factor activity, protein tyrosine kinase activity, transmembrane receptor protein tyrosine kinase activity, etc.; BP: phosphatidylinositol 3-kinase signaling, protein autophosphorylation, regulation of phosphatidylinositol 3-kinase signaling, regulation of inflammatory response, positive regulation of lipid metabolic process, etc.; CC: lipid rafts, membrane microdomains, nuclear envelope lumen, plasma membrane cytoplasmic side, endoplasmic reticulum lumen, etc., as shown in Figures 5A–C. In terms of pathway enrichment analysis, this study selected pathways related to metabolic processes based on Reactome pathway enrichment results. The results showed that there are eight pathways related to lipid metabolism: regulation of lipid metabolism by PPARα, SREBF activates gene expression, steroid hormone metabolism, synthesis of EPA-derived SPMs, synthesis of DPA-derived SPMs, triglyceride metabolism, arachidonic acid metabolism, and synthesis of DHA-derived SPMs, as shown in Figure 5D. Figure 6A is the Reactome lipid metabolism pathway enrichment chart. Figure 6B summarizes the chord diagrams of the four key pathways selected after combining literature analysis. The key pathways include regulation of lipid metabolism by PPARα, SREBF activates gene expression, steroid hormone metabolism, and arachidonic acid metabolism.

### 3.5 Molecular docking

**FIGURE 7:**
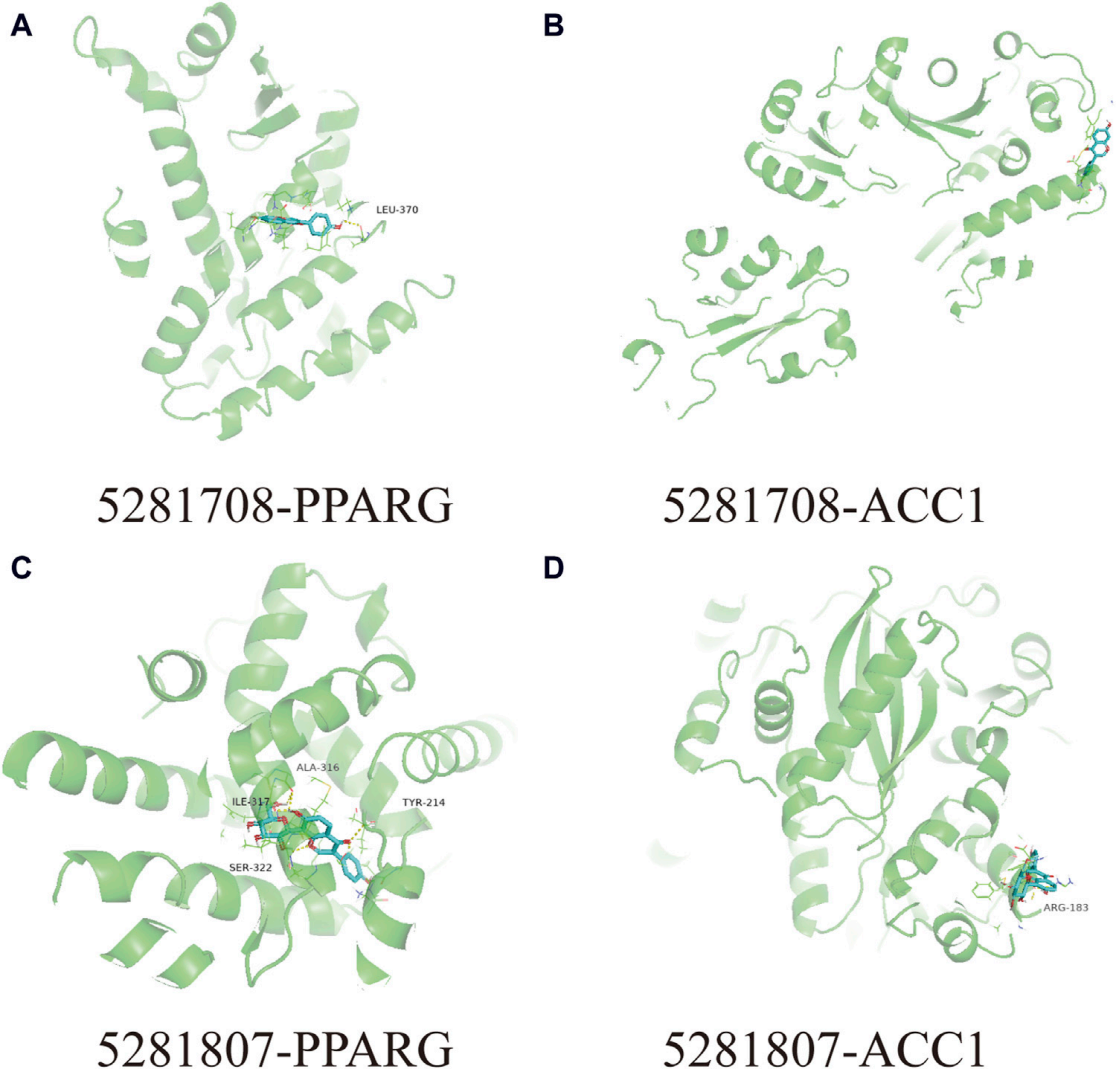
Analyze the docking situation. **(A)** 5281708-PPARG, **(B)** 5281708-ACC1, **(C)** 5281807-PPARG, **(D)** 5281807-ACC1.

In this docking, two Puerariae Lobatae Radix chemical components were selected, namely, Daidzein, and Puerarin. The pathways selected for docking were Regulation of lipid metabolism by PPARα, Activation of gene expression by SREBF and Metabolism of steroid hormones. A total of 12 potential pathway proteins that Puerariae Lobatae Radix might intervene with were collected and docked. The two key components of Puerariae Lobatae Radix have good docking relationships with five pathway targets: ACC1, PPARG, PPARA, FABP1, and ALOX5 (docking energy < −7.0). The molecular docking results are shown in Table 2. We further drew molecular docking simulation diagrams of Puerarin and Daidzein with PPARG and ACC1, as shown in Figures 7A–D.

**TABLE 2:** Docking information of Puerariae Lobatae Radix with key pathway targets.

Pathway	Gene	Daidzein (5281708)	Puerarin (5281807)
Activation of gene expression by SREBF	ACC1	−7.5	−8.1
Regulation of lipid metabolism by PPARalpha	PPARG	−7.6	−7.5
	PPARA	−8.1	−8.7
	FABP1	−7.9	−8.8
Metabolism of steroid hormones	ALOX5	−8	−8.7

### 3.6 Animal experimental validation

The golden hamster has abundant hair follicles and sebaceous glands on both sides of the back, and its sebaceous gland biological characteristics are similar to humans. During the breeding process, the area of the sebaceous gland patches on both sides of its back will continuously increase, which can evaluate the changes in the area of the sebaceous gland patches on both sides of the golden hamster’s back before and after the intervention of Puerariae Lobatae Radix.

#### 3.6.1 Comparison of sebaceous gland patch areas in each group

**FIGURE 8:**
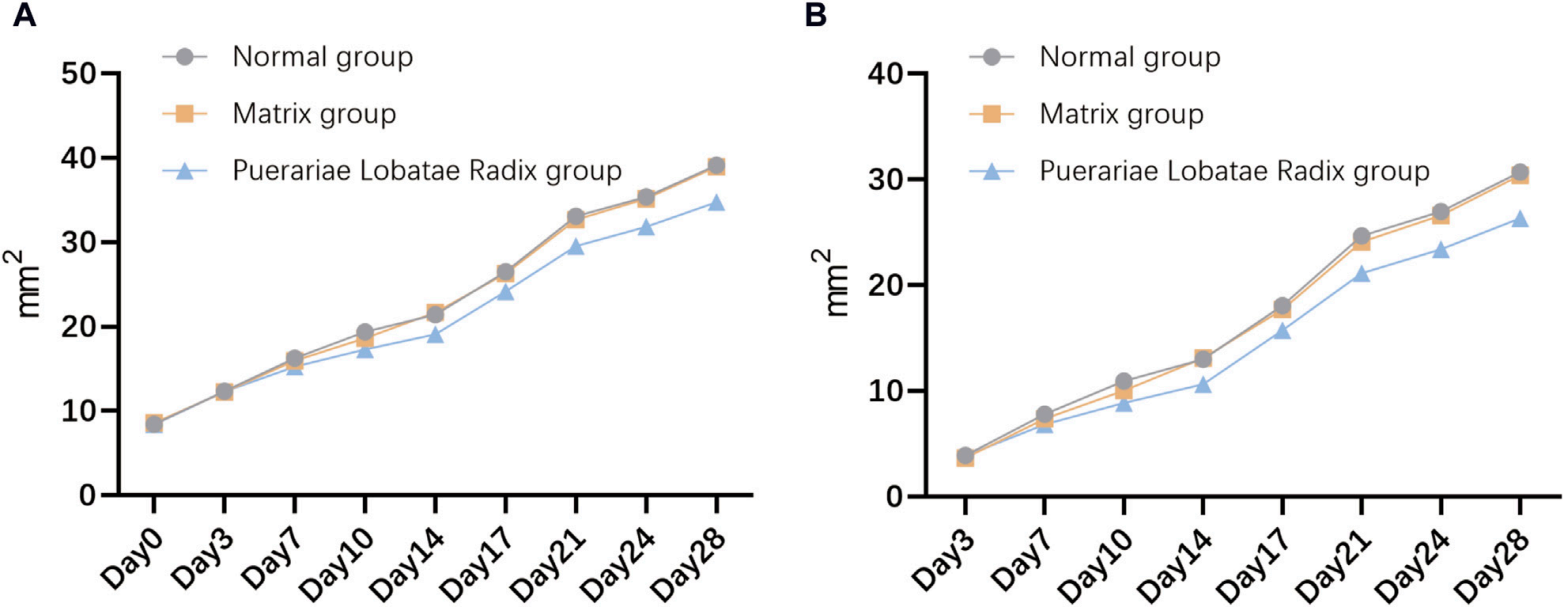
**(A)** Effects of Puerariae Lobatae Radix on the sebaceous gland spot area on both sides of the belly of golden hamsters. **(B)** Increased area at each time point compared to before administration.

The results of the influence of Puerariae Lobatae Radix on the sebaceous gland patch area on both sides of the golden hamsters’ back are shown in Figure 8A. From the results, it can be seen that there is no significant difference in the sebaceous gland patch area of golden hamsters in the blank control group, matrix group, and Puerariae Lobatae Radix group before administration. From the third day, the area of the blank group and matrix group increased significantly compared to the Puerariae Lobatae Radix group, but there was no significant difference between the blank group and matrix group. The results of the increased area compared to before administration at each time point are shown in Figure 8B. From the results, it can be seen that on the third day, there was no significant difference in the increased sebaceous gland patch area of golden hamsters in the blank control group, matrix group, and Puerariae Lobatae Radix group before administration. After the third day, the areas of the blank group and matrix group increased significantly compared to the Puerariae Lobatae Radix group, but there was no significant difference between the blank group and matrix group.

#### 3.6.2 Pathological changes in sebaceous gland patch tissue

**FIGURE 9:**
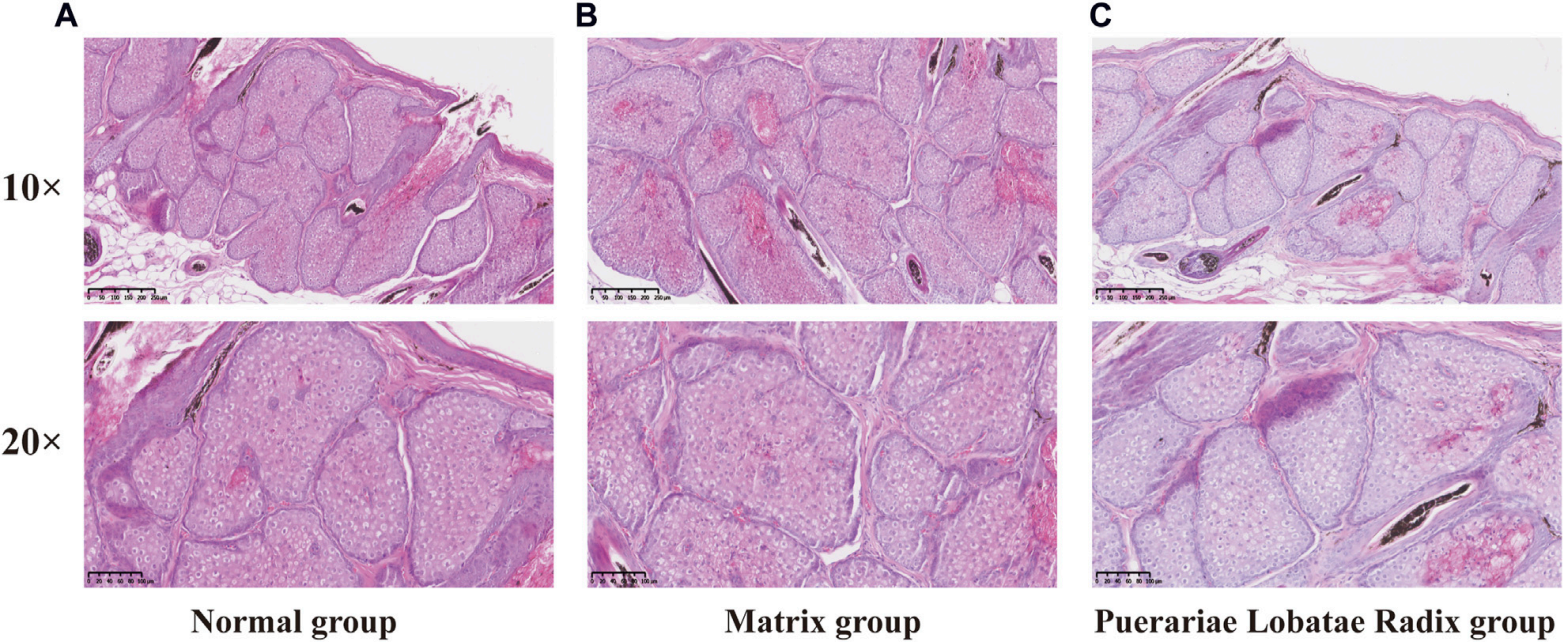
**(A)** normal group, **(B)** Matrix group and **(C)** Puerariae Lobatae Radix group. Each group was observed by 10-fold and 20-fold skin histopathological HE staining.

The results of HE staining of the skin tissue sebaceous gland structure of the golden hamsters in each group are shown in Figure 9. From the HE staining, it can be seen that in the blank control group and matrix group, the sebaceous glands of the golden hamsters are multi-layered and lobulated, with full and numerous glands that are closely and thickly arranged, indicating that the matrix does not affect the normal growth of the sebaceous glands in the experimental animals. Compared to the blank control group and matrix group, the sebaceous gland structure of the golden hamsters in the Puerariae Lobatae Radix group has obvious changes, with most of the sebaceous glands being smaller, thinner, and more loosely arranged.

#### 3.6.3 Effects of Puerariae Lobatae Radix on TG, DHT, IL-6

**FIGURE 10:**
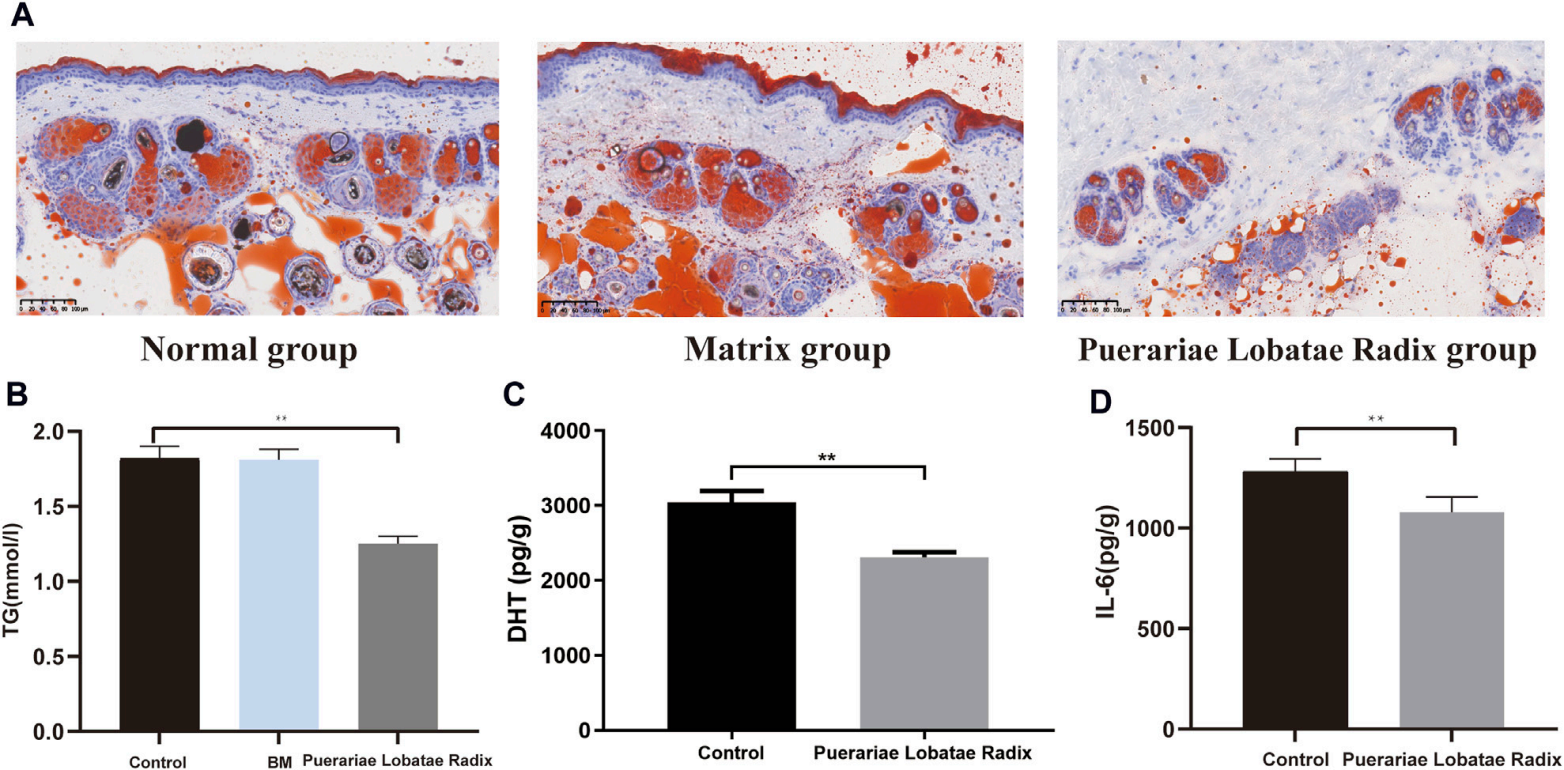
**(A)** Oil red staining results. **(B–D)** Effects of Puerariae Lobatae Radix on the expression of TG, DHT, and IL-6 in the sebaceous glands of golden hamsters. (**p < 0.01, compared with the blank control group).

Oil Red staining shows that, compared to the control group and matrix group, there is less lipid deposition in the sebaceous glands of the golden hamsters in the Puerariae Lobatae Radix group, Figure 10A. The effect of Puerariae Lobatae Radix on TG in the sebaceous gland tissue of golden hamsters is shown in Figure 10B. The effects on DHT and IL-6 are shown in Figure 10C,D. From the results, it can be seen that there is little difference in the TG indicator results in the sebaceous gland tissue of the golden hamsters in the blank control group and matrix group. The biochemical indicator results also verify that the matrix used in this experiment does not affect the normal growth of the golden hamster’s sebaceous glands. Compared to the blank control group, Puerariae Lobatae Radix significantly reduces the expression of TG, DHT, IL-6 (*p* < 0.01)

#### 3.6.4 Effects of Puerariae Lobatae Radix on PPARG and ACC1

**FIGURE 11:**
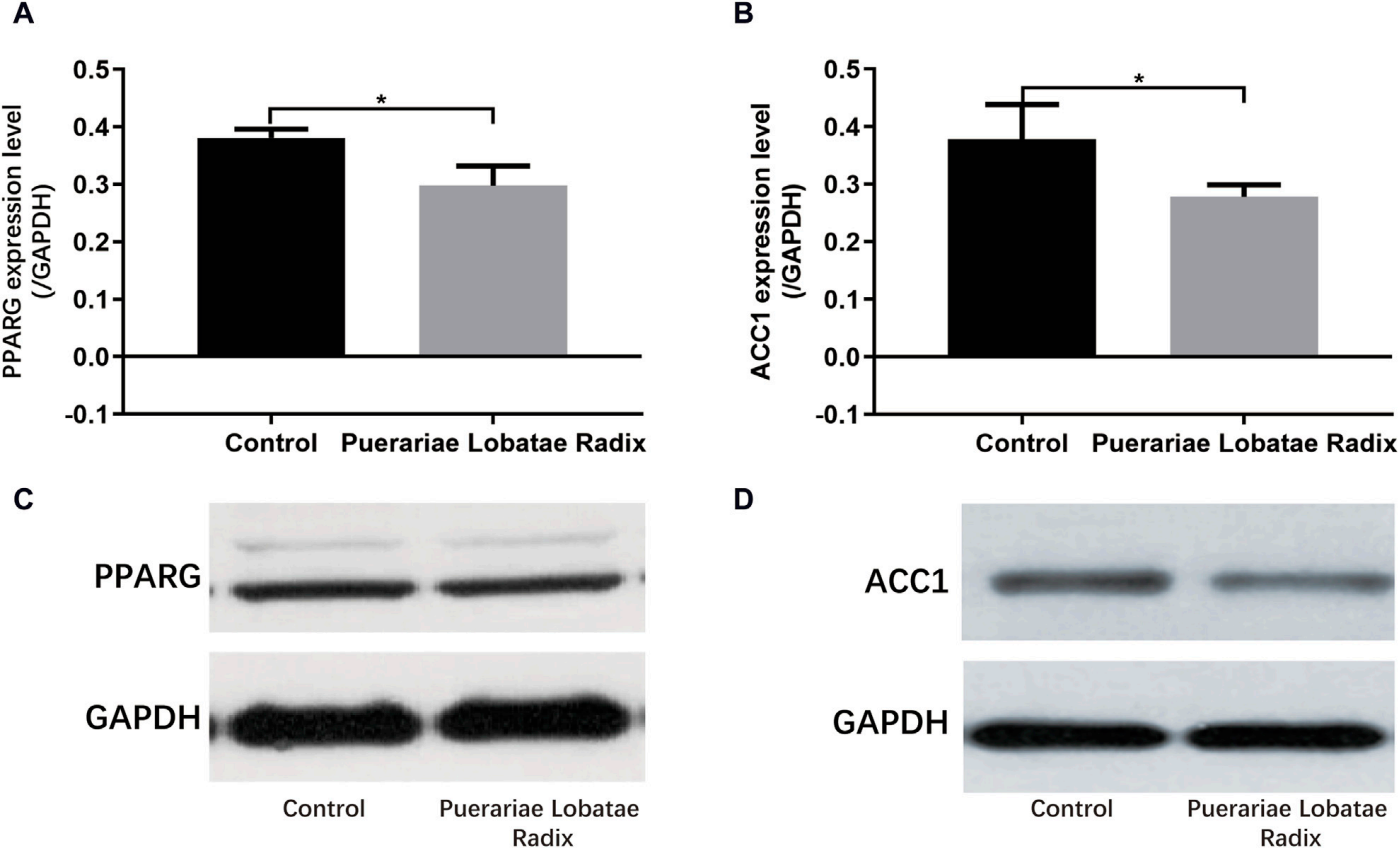
**(A–D)** Effects of Puerariae Lobatae Radix on the expression of PPARG and ACC1 in the sebaceous glands of golden hamsters (*p < 0.05, **p < 0.01, both compared with the blank control group).

PPARG is a key regulatory factor for lipogenesis. ACC1 is a downstream target gene of SREBP. Once the SREBP protein is activated, it promotes the synthesis and secretion of lipids in the sebaceous glands. The activation of these key factors results in a higher content of components like TG in the sebum, leading to hyperkeratosis and incomplete keratinization, and even skin inflammatory reactions. Western blotting results showed Puerariae Lobatae Radix significantly decreases the expression of PPARG, ACC1 (*p* < 0.05), as shown in Figure 11.

## 4 Discussion

The malfunction of sebaceous glands is a significant contributing factor to the development of acne, seborrheic dermatitis, and various other dermatological conditions. The accumulation of excessive sebum combined with keratinocytes within hair follicles can facilitate the formation of comedones, such as blackheads and whiteheads. Additionally, the abundance of sebum can serve as a favorable nutrient source for cutaneous bacteria, particularly Propionibacterium acnes. The proliferation and metabolic byproducts of these bacteria may induce inflammation, thereby contributing to the development of acne. Furthermore, elevated levels of free fatty acids in sebum can trigger skin irritation and seborrheic dermatitis. Puerariae Lobatae Radix has a spicy and cool taste, which can dispel external pathogens from muscles and skin. Its therapeutic effects play a significant role in skin diseases. In clinical practice, Puerariae Lobatae Radix is commonly used to treat acne and seborrheic dermatitis, but its mechanism of action remains unclear. This study, combining network pharmacology with animal experiments, explores the regulatory mechanism of Puerariae Lobatae Radix on sebaceous gland secretion.

Network pharmacology research found that the primary components of Puerariae Lobatae Radix regulating sebaceous gland secretion are Puerarin and Daidzein. Puerarin is an isoflavonoid compound extracted from the root of Puerariae Lobatae Radix, with a wide range of pharmacological effects. It is currently clinically used for lowering blood pressure (Wu et al., 2020), delaying the progression of cardiac hypertrophy (Liu et al., 2015), improving myocardial fibrosis (Zhang et al., 2011), lowering blood lipids, inhibiting coronary artery disease thrombosis formation (Deng et al., 2017), lowering blood sugar and improving insulin resistance, anti-tumor (Zhang et al., 2018), anti-oxidation (Hwang and Jeong, 2008), anti-inflammatory, and also has plant estrogen-like effects. Daidzein is essentially an isoflavone and belongs to the flavonoid class, proven to have various pharmacological activities, including anti-cancer, anti-inflammatory, cardiovascular disease prevention, and cholesterol reduction. Animal experiments also confirmed the anti-inflammatory effects of Puerariae Lobatae Radix. Combined with Reactome pathway enrichment results and literature analysis, the most critical pathways for Puerariae Lobatae Radix to regulate sebaceous gland secretion were found to be pparα lipid metabolism regulation, SREBF gene expression activation (SREBP), steroid hormone metabolism, and arachidonic acid metabolism. Therefore, this study’s animal experiment phase mainly explored the pparα and SREBP pathways.

Animal experiments have shown that Puerariae Lobatae Radix can reduce the expression of TG, DHT, IL-6, PPARG and ACC1 in the sebaceous gland tissue of golden hamsters. TG is one of the components of sebum secreted by the sebaceous gland. Therefore, regulating and inhibiting TG’s lipid metabolism plays a crucial role in reducing or slowing down abnormal sebaceous gland lipid secretion. This study also confirmed this, as Puerariae Lobatae Radix can reduce TG expression in the sebaceous gland, thereby decreasing sebaceous gland secretion. An increase in lipid secretion from the sebaceous gland can cause skin inflammation, a prerequisite for the development of common acne (Clayton et al., 2019). IL-6 is an important inflammatory factor in the development of diseases such as common acne and seborrheic dermatitis. Some findings suggest that IL-6 *in vitro* can stimulate SZ95 cell lipid synthesis (Hu et al., 2016), indicating that IL-6 may also be involved in the occurrence of diseases like common acne by regulating the biological function of sebaceous gland cells. It can upregulate lipid synthesis-related genes PPARG, FAS, and SREBP1, suggesting that IL-6 can promote lipid synthesis in sebaceous gland cells. Thus, reducing IL-6 plays a pivotal role in regulating lipid synthesis and alleviating diseases caused by excessive sebum secretion. There are also studies showing that DHT can affect lipid metabolism by regulating the activity of PPARs (Singh et al., 2003; Sato et al., 2013).

**FIGURE 12:**
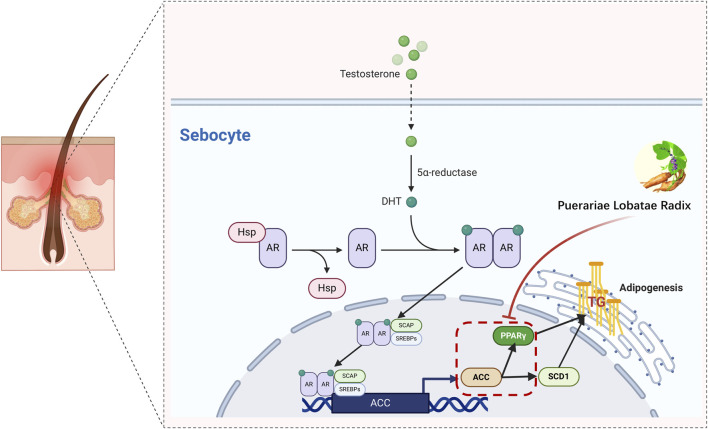
Simulation of a potential mechanism by which Puerariae Lobatae Radix regulates sebaceous gland secretion.

The reduction in TG is also related to PPARG and ACC1, where PPAR-γ is a key regulator of fat generation (Morán-Salvador et al., 2011). Its reduced expression activity can improve lipid metabolism in the body and lower TG levels (Yamauchi et al., 2001). Studies have shown that the AMPK-ACC pathway can inhibit TG synthesis in the body (Yuan et al., 2018). Activation of these two pathways collectively reduces TG levels. PPARG is a member of the peroxisome proliferator-activated receptor (PPAR) nuclear receptor subfamily, encoding the protein PPARG, a regulator of adipocyte differentiation. PPARG has been proven to regulate the differentiation and maturation of fat cells, and its increased expression promotes the body’s absorption and deposition of lipids (Barber and Travers, 1995). Additionally, PPARG has been involved in the pathology of many diseases, including obesity, diabetes, atherosclerosis, and cancer. It controls the peroxisome oxidation pathway of fatty acids. It is a key regulator of adipocyte differentiation and glucose homeostasis (Rodríguez-García et al., 2022), promoting sebum synthesis secretion by inhibiting the WNT signaling pathway, and promoting the differentiation and proliferation of the sebaceous gland (Zhang et al., 2006). Excessive sebaceous gland secretion is often diagnosed as phlegm dampness in traditional Chinese medicine, and some research indicates that the downregulation of PPAR-γ gene expression in metabolic syndrome phlegm syndrome patients may be the biological basis of its phlegm syndrome complexity (Lili et al., 2015). In summary, the PPAR pathway has a certain association with TCM phlegm syndrome. This study screened out seven main components of Puerariae Lobatae Radix through network pharmacology and found that six of them can dock well with PPARG through molecular docking. According to the docking energy, the molecular docking of Puerarin and PPARG is the most stable. Therefore, we further verified the regulatory effect of Puerariae Lobatae Radix on PPARG through animal experiments, and the results showed that the Puerariae Lobatae Radix group effectively downregulated the expression of PPARG in the sebaceous gland, indicating that Puerariae Lobatae Radix might regulate the PPAR pathway through PPARG, thereby affecting the lipid metabolism ofmouse sebaceous glands in Figure 12.

The function of the sebaceous gland is regulated by androgens. DHT, as the most effective androgen (Duarte et al., 2019), binds to AR to stimulate the proliferation of sebaceous gland cells. Once AR is activated and binds with ARE, it accelerates the transcription of SCAP, promoting the activation of SREBPs. The SREBP-1 signaling pathway is one of the vital routes regulating lipid synthesis signal transduction. SREBPs belong to the nuclear transcription factor family and are crucial nuclear transcription factors regulating lipid metabolism (Siculella et al., 2016). They can directly activate the expression of several genes related to the synthesis and uptake of triglycerides and fatty acids. There are three isoforms of SREBPs in mammals, namely, SREBP-1a, SREBP-1c, and SREBP-2 (Cheng et al., 2018). SREBP-2 primarily regulates cholesterol metabolism, while SREBP-1 is involved in the synthesis of cholesterol and fatty acids. Some research suggests that daidzein can dose-dependently increase the expression and maturation of SREBP-1c. ACC is one of the downstream target genes of SREBP-1 (Lee et al., 2017; Hong et al., 2019) and is the rate-limiting enzyme controlling the first step of fatty acid biosynthesis (Barrault et al., 2015). Reducing ACC1 can effectively slow down lipid synthesis. Some studies show that soy isoflavones can significantly downregulate ACC1 expression (Kuhn et al., 2004).

In this study, only single targets were selected in the pathway for lateral research, and experiments were solely conducted from an animal perspective. Future research will select multiple targets for longitudinal study in each pathway and conduct experimental research at the cellular level to better reveal the mechanism of Puerariae Lobatae Radix in inhibiting sebaceous gland secretion.

## 5 Conclusion

Through network pharmacological prediction and subsequent animal experiments, our study suggests that Puerariae Lobatae Radix exhibits the potential to regulate and suppress sebaceous gland growth in golden hamsters. Additionally, it can reduce the secretion of TG from these glands and mitigate the expression of DHT and IL-6. This effect may be attributed to the modulation of lipid-related pathways by downregulating the expression of PPARG and ACC1. Notably, Puerarin and Daidzein, two constituents found in Pueraria root, appear to play pivotal roles in this process. These findings hold promise for the enhanced clinical utilization of Pueraria, offering a novel approach to addressing abnormal sebaceous gland secretion.

## Data Availability

The data presented in the study are deposited in the jianguoyun repository, available at: https://www.jianguoyun.com/p/DSsaktUQ1LLSDBirssYFIAA.
